# Game-based learning approach on students’ motivation and understanding of chemistry concepts: A systematic review of literature

**DOI:** 10.1016/j.heliyon.2022.e09541

**Published:** 2022-05-27

**Authors:** Edwin Byusa, Edwige Kampire, Adrian Rwekaza Mwesigye

**Affiliations:** a African Centre of Excellence for Innovative Teaching and Learning Mathematics and Science (ACEITLMS), University of Rwanda College of Education (URCE), Kayonza, P.O Box: 55, Rwamagana, Rwanda; b University of Rwanda College of Education (URCE), Kayonza, P.O Box: 55, Rwamagana, Rwanda; c Department of Educational Foundation and Psychology, Mbarara University of Science and Technology (MUST), Uganda

**Keywords:** Activity-based learning, Chemistry concepts, Chemistry education, Game-based learning, Secondary school, Teacher and learner-centered pedagogy

## Abstract

The past decade has seen a significant shift from teacher-centered pedagogy to a learner-centered approach in chemistry education research. Game-based learning has emerged as one of the most beneficial instructional approaches because it emphasizes “hands-on” and “minds-on” activities in chemistry classrooms. However, there has been a scarcity of review studies in chemistry education research that have attempted to document different educational games implemented and how such games have contributed to enhancing students' motivation and understanding of chemistry concepts. A total of 57 articles were reviewed to identify educational games implemented in chemistry classrooms from 2010 to 2021 to address this gap. All the reviewed articles were downloaded from the Google Scholar search engine and have all been indexed by Scopus. A systematic analysis was adopted to identify the purposes, educational game designs and implementation, and the chemistry content areas of focus for all the reviewed studies. Results show that educational games enhance students’ conceptual understanding of chemistry and increase their motivation to learn and have fun while making sense of the learned content.

## 1 Introduction

Like other physical and natural sciences, chemistry occupies a central position in daily living as it provides affordances for learners to understand the environment around them. However, chemistry teaching and learning at all levels of education has been marked with several challenges. For instance, students’ lack of problem-solving skills, limited spatial visualization, difficulties in understanding chemistry vocabulary, and poor communication between students and teachers have been previously studied (Childs and Sheehan, 2009; Erman, 2017). This has prompted researchers from different settings to continuously advocate for a learning environment that would appropriately address such difficulties.

The teacher-centered pedagogy is characterized by the teacher's explanations of content, demonstration of experiments, and limited interactions among students or between students and the teacher (Eilks et al., 2013; Nzeyimana and Ndihokubwayo, 2019). This teacher-centered teaching approach is based on the behaviorist point of view, which suggests that giving the correct information to the students enables them to commit such information to memory, assign appropriate meaning, and have it ready to be used in the future (Mills, 2000). In this way, chemistry learning is characterized by students' rote learning. However, the present understanding of meaningful learning in chemistry classrooms has been attributed to learner-centered, active, and cooperative learning approaches (Cahyana et al., 2017; Kara, 2021). These approaches to learning stem from the theory of social cognitive and constructivism. The social constructivist learning approach is premised on the notion that meaningful learning can only occur if learners are provided with opportunities to interact as they attach meaning to the learned content (Amineh and Hanieh, 2015). Cognitivists concur that learners pack the mind and understand the content through useful hands-on and mind-on activities (Yilmaz, 2011), such as laboratory activities, animations, computer simulations, and videos (Ndihokubwayo et al., 2020). In fact, there was a need to shift from teacher-centered to learner-centered because it has been proved that learners learn well when they are given the opportunity to learn with their peers instead of sitting passively in their class and listening to the teacher. To achieve this, active learning techniques such as the cooperative learning approach (Sibomana et al., 2021), game-based approach (North et al., 2021), problem-solving approach (Dorimana et al., 2021), and others should be brought to the classroom.

This is why instructional approaches in line with the social constructivist view of learning have been widely used in chemistry classrooms at all levels of education. Instructional approaches such as activity-based learning, cooperative learning, and game-based learning, among others, have been found effective in the teaching and learning of chemistry concepts (Byusa et al., 2020; da Silva Júnior et al., 2021; Eilks et al., 2013; Prins et al., 2016). This has been attributed to the fact that such learning approaches are bound to engage students physically, socially, and cognitively. This eventually brings about a positive attitude, increased interest, and motivation towards learning chemistry. Constructivism connects to games in learning as students are equipped with useful activities to build learning from their experience (Bhattacharjee, 2015).

Although the definition of game-based learning is somewhat ambiguous due to the variety of formats and contexts in which it has been applied. The bottom line is that educational games do not only enhance students’ conceptual understanding but also increase their motivation to learn and allow them to have fun while making sense of the learned content (Baek et al., 2015; da Silva Júnior et al., 2021; Franco-Mariscal et al., 2016; Partovi and Razavi, 2019). Students' understanding is the students grasping a certain concept, fully and scientifically understanding the meaning and usability of that concept in real life. Despite all the benefits that have been highlighted about the game-based approach in chemistry education research, its implementation remains challenging for many teachers, especially in the less developed world. For instance, our previous study revealed that teachers rarely use games in their daily teaching activities (Byusa et al., 2020) while they proved effective in students learning (Rahman et al., 2020).

While several review studies have been conducted on game-based learning (Cahyana et al., 2017), most have generally focused on science. Furthermore, few have narrowed it down to specific content areas of chemistry. In other words, there has been a scarcity of review studies in chemistry education research that have attempted to document different educational games that have been implemented in chemistry classrooms and how such games have contributed to the enhancement of students’ understanding of chemistry concepts or motivation to learn. With this background, we attempted to review different educational games and their contribution to chemistry teaching and learning across all school levels of education. In that regard, our review was guided by the following research questions:

i. What are some of the game-based learning techniques that have been employed in chemistry classrooms in the last ten years?
ii. What were the chronological order, source countries, educational level, and delivery modes of the identified game-based learning techniques?
iii. How could each of the identified game-based techniques contribute to students' motivation and understanding of secondary school chemistry concepts?

## 2 Methodology

Data to address the already-stated research questions were collected via a systematic review method. This was done by conducting a literature search to gather the published work on the effectiveness of game-based learning in the teaching and learning of chemistry. We used keywords such as game-based learning, activity-based learning, physical embodiment, cognitive embodiment, and collaborative or active learning in chemistry classrooms in the google scholar search engine. Initially, 83 documents were downloaded, including three book chapters, two electronic books, 13 conference papers, and 65 peer-reviewed journal articles. Our literature search was also restricted to the work published from January 2010 to November 2021.

After doing some preliminary analysis of the downloaded literature, we had to exclude some documents because of at least one of the following reasons:

• The paper is not related to chemistry
• The paper has been published in a journal not indexed by Scopus
• The game-based approach employed is not clear
• The paper does not provide enough information that is needed for addressing the research questions.

**Figure 1:**
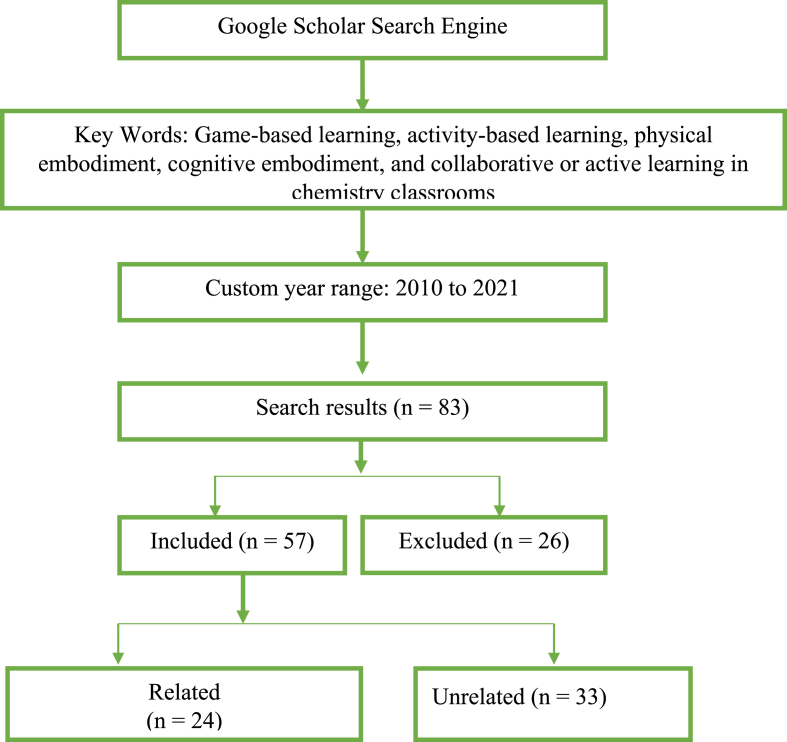
Article selection process.

Using a similar procedure as the one employed by Kara (2021) and Ukobizaba et al. (2021), a summary of the article selection process is illustrated in Figure 1.

Using the above-prescribed criteria, 57 peer-reviewed articles from reputed journals were deemed suitable for review. Thus, 26 documents—book chapters, electronic books, proceedings, and unreputed journal articles (from predatory or local journals)—were excluded. This means that 26 documents were substracted from 83 and 57 remained. It should also be noted that “reviewed papers” do not constitute all the references because other works were only cited for providing evidence of some claims in the discussion of research findings.

Although we reviewed 57 articles related to game-based learning, some of the selected papers did not mention games (a certain game used was not clear) or related to chemistry. Therefore, we excluded them and retained 24 articles that clearly show an implemented game in teaching and learning chemistry. Thus, we excluded 33 articles. Using manual sorting, we identified the purposes, educational game designs and their implementation, and the chemistry content areas of focus for all the reviewed studies. At the same time, an Excel spreadsheet assisted in descriptive analysis. We identified the game used in the article, its purpose, delivery mode, educational level, year of publication, country of the study, and the chemistry concept catered for.

## 3 Findings

**RQ1. What are some of the game-based learning techniques that have been employed in chemistry classrooms in the last ten years?**

Table 1 displays the name of the game/approach, its purpose, and the delivery mode. It also contains the study site (where the study was conducted), education level, and chemistry concepts. The purpose shows the author(s) and the year of publication. Based on the results displayed in Table 1, it can be inferred that fourteen (24) games or game-based approaches were identified from 2010 till 2021. These games are activity cards, element cycles, card, and computer games, board games, legends of Alkhimia, 3D Role-playing Game (3D RPG), game-based approach, Chemory game, picture-Chem, Chairs!, mobile augmented reality (AR) application, Misha and Kosha game-based learnings, chemical nomenclature, Molebots, Ion Hunters, ChemEscape, card game, and bioplastic synthesis activity, Pantomime MisCoAct, ChemDraw, cooperative games, ORG600, WIL, CheMakers, and ABSQR Code Game.

**Table 1:** Identified games/game-based approaches for chemistry classrooms.

	Purpose of the study	Country	Game	Delivery mode	Education level	Chemistry concepts
1	Observing students' understanding of the “Elements and Compounds” unit (Duvarci, 2010)	Turkey	Activity cards	Non-digital	Secondary school	Elements and Compounds
2	Reinforcing the correlation of essential elements and their different forms in the ecosystem (Pippins et al., 2011)	United States	Element Cycles	Non-digital	High school	Four ecosphere sections
3	Investigate the effect of teacher-made instructional card games and computer games on the learning of chemistry concepts in high school students (Rastegarpour and Marashi, 2012)	Iran	Card games and computer games	Both digital and non-digital	High school	Chemistry
4	Design and Implementation of an Educational Game for Teaching Chemistry in Higher Education (Antunes et al., 2012)	Brazil	Board-game	Non-digital	University	Molecular geometry, polarity, and intermolecular forces
5	Fostering chemistry teaching and learning through inquiry (Chee and Tan, 2012)	Singapore	Legends of Alkhimia	Digital	Secondary school	Mixtures, solutes, and immiscible liquids
6	Improving students' performance and motivation in learning chemical formulas (Chen et al., 2014)	Taiwan	3D Role-playing Game (3D RPG)	Digital	Secondary school	Chemical formula
7	Chemistry teaching and learning through the use of games as educational tools (Wilson and Samide, 2014)	United States	Game-Based Approach	Both digital and non-digital	University	Analytical and Organic Chemistry
8	Increase student motivation and achievement for an undergraduate physical chemistry course (Daubenfeld and Zenker, 2015)	Germany	Chemory game	Digital	University	Physical chemistry
9	Learning of common laboratory apparatus in chemistry (Kavak and Yamak, 2016)	Turkey	Picture Chem	Non-digital	High school graduates	Chemistry laboratory equipment
10	Teaching and learning of the ring flip of cyclohexane (Winter et al., 2016)	United States	Chairs!	Digital	Undergraduate	Cyclohexane
11	Evaluate the effectiveness of using mobile augmented reality (AR) instructional material for Year 10 science students in a secondary school in Brunei Darussalam (Wan et al., 2018)	Brunei	Mobile augmented reality (AR) application	Digital	Secondary school	Redox reaction
12	Evaluate the effectiveness of Game-Based Learnings on Elementary School Students' achievement (Partovi and Razavi, 2019)	Iran	Misha and Kosha Game-Based Learnings	Digital	Elementary	Elementary science
13	Review chemical nomenclature in an engaging and fun way on their own by answering random questions from a database with over 700 questions (Sousa Lima et al., 2019)	Brazil	Chemical Nomenclature	Digital	High school and undergraduate students	Chemical Nomenclature
14	Implementations of Molebots and other media (Gupta, 2019)	United States	Molebots	Digital	High school and university	Chemical Nomenclature
15	Strengthening students' abilities in identifying the names and formulas of anions and cations (Yenikalaycl et al., 2019)	Turkey	Ion Hunters	Non-digital	Undergraduate	Naming anions and cations and
16	Engagement and enhancement of problem-solving skills and hands-on experiences (Clapson et al., 2019)	Canada	ChemEscape	Non-digital	Grade 4–12 and first-year undergraduate level	Laboratory activities
17	Communicate key concepts using multiple communication methods (visual, verbal, acting) (Clapson et al., 2020)	Canada	Card game and bioplastic synthesis activity	Non-digital	Grades 5–8, Second-year engineering students	Materials and Polymer Chemistry
18	Develop a game for pre-service teachers on typical students' misconceptions using Participatory Action Research (Belova and Zowada, 2020)	Germany	Pantomime MisCoAct	Non-digital	High school teachers	Nature of matter, Acids and Bases, Electrochemistry, Chemical bonding, equilibrium, and reactions
19	Improving students' wellness and organic chemistry skills during the COVID-19 Outbreak (Fontana, 2020)	United States	ChemDraw	Digital	High school, Undergraduate	Chemistry laboratory
20	Time effect of cooperative games on students' emotions of learning science and the treatment effect on their chemistry achievement (Chen et al., 2020)	Taiwan	Cooperative Games (such as cads, board games, and riddles)	Both digital and non-digital	High school	Basics of chemical elements and compounds
21	Individually review organic reactions in an engaging and fun way by answering over 600 multi-choice questions (da Silva Júnior et al., 2021)	Brazil and France	ORG600 (organic reactions game)	Digital	University	Organic reactions
22	Enhancing student employability (Ponikwer and Patel, 2021)	United States	Work-integrated learning (WIL)	Digital	University	Chromatography
23	Enhancing students' understanding of organic reaction mechanisms and developing mechanistic thinking skills (Zhang et al., 2021)	Singapore	CheMakers	Non-digital	Undergraduate	Organic chemistry
24	Facilitating conceptual understanding of the names, formulas, types, and reactions of acids, bases, and salts among high school and university students (Harman and Yenikalayc, 2020)	Turkey	ABSQR Code Game	Digital	High school and university students	Acids, bases, and salts

The reviewed literature also shows that most of these educational games were applied on teaching topics such as elements and compounds, organic chemistry, organic reaction mechanisms, names and formulas of anions and cations, chemical formulas, acids, bases, and salts, lab apparatus, and chemical elements, among others (see Table 1).

**RQ2. What were the chronological order, source countries, educational level, and delivery modes of the identified game-based learning techniques?**

**Figure 2:**
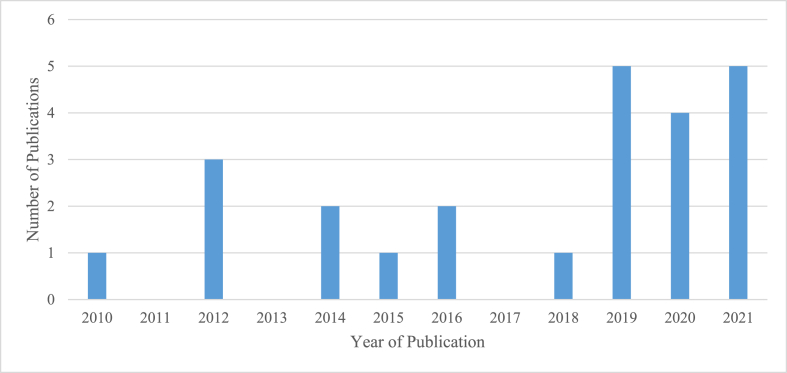
Chronological order of publication for games developed and used to teach chemistry.

Regarding the chronological order of publication for games developed and used to teach chemistry, one article was published in 2010, another in 2011, and three in 2012, as Figure 2 presents (for the specificity of game, see Table 1 above). Many games were developed and implemented from 2019, where each year got more than three publications. Note that each dot represents a published article that developed or used an educational game in a certain year.

**Figure 3:**
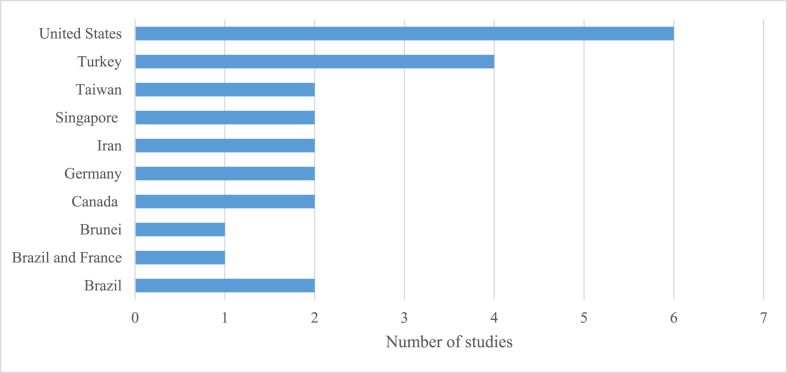
Count of studies related to chemistry games conducted in various countries across the globe.

Like the chronological order of implemented games in teaching chemistry, Figure 3 shows the number of studies corresponding to their study country. For instance, the United States has produced many studies as it got six (25%) publications from 2010. Africa and Oceania got 0%, South America got 13%, while Asia, Europe, or North America got 29%.

**Figure 4:**
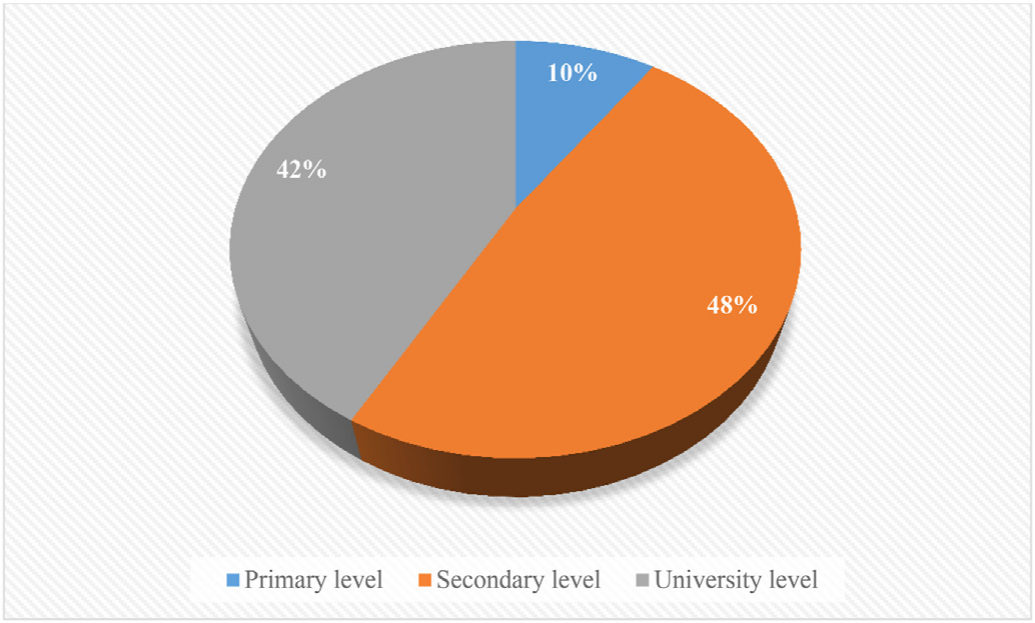
Rate of games implemented across educational levels.

From the chronological order and country of origin, it was found that the partition or implementation of these games in university or secondary school is quite the same (see Figure 4). For instance, only three studies (10% of studies) were done in elementary or primary school. Fifteen (48%) studies or games were done or implemented in secondary or high school, while 13 (42%) studies or games were done or implemented in university. The total does not add up to 24 analyzed studies because some were done in more than one level.

**Figure 5:**
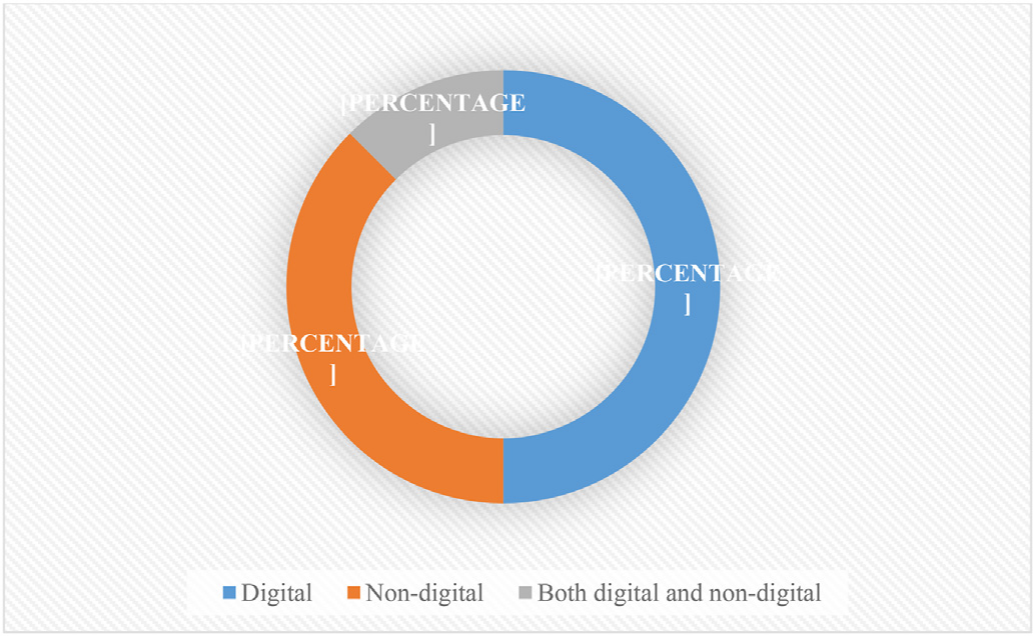
Delivery modes of the identified educational games.

Regarding the second research question, research findings show that some of the identified games were delivered through the use of information, and communications technology (ICT) tools (digital games). In contrast, others did not involve any use of ICT gadgets or tools (non-digital games). While Figure 5 illustrates the proportion of digital, non-digital, and both digital and non-digital games, Table 1 shows the purpose and delivery mode (digital or non-digital) for each identified educational game. Based on the results displayed in Figure 5, it suffices to point out that the number of non-digital games (n = 12, or 50%) was not substantially different from that of digital games (n = 9 or 37%). Only three (13%) of the identified games had been implemented both in both digital and non-digital modes.

To provide answers to the third research question and compliment the previous research questions, significant findings and future directions for the identified game-based approaches are discussed in the following sub-headings.

## 4 Discussion

**RQ3. How could each of the identified game-based techniques contribute to students’ motivation and understanding of secondary school chemistry concepts?**

The third research question relates to the study's main aim on the game-based learning techniques in secondary school students' motivation and understanding of chemistry concepts. To provide answers to this research question, each of the identified game-based learning approaches is discussed in terms of how it affects students' learning of chemistry concepts. In addition, the main proponents and the context for each identified game-based learning approach have also been highlighted.

### 4.1 3D role-playing game (3D RPG)

Regardless of the type of exploratory strategy employed, learners showed mild positive motivation towards learning chemistry (chemical formula concepts in particular) via a 3D role-playing game (3D RPG). This game was implemented in a study conducted by Chen et al. (2014) in which one hundred and fifteen (115) eighth-grade students from a Taiwanese school voluntarily participated in the 3-week experiment. These findings provide evidence on the need to engage learners in classroom activities that are bound to offer opportunities for them to make use of the acquired knowledge and skills that would eventually increase their motivation to learn.

### 4.2 ABSQR code game

Harman and Yenikalayc (2020) administered the ABSQR Code Game to 15 students in three groups for three rounds. These students were drawn from the first-year science teaching program. Research findings revealed that students developed a positive opinion about using games in chemistry teaching and learning. It was further stated that the ABSQR Code game enabled students to learn in a fun but helpful manner. While the game was trialed on tertiary level students, the game is applicable at the secondary school level since secondary students also learn about acids, bases, and salts.

### 4.3 Activity cards

This approach was implemented by Duvarc (2010) in a chemistry class of grade 9 students. This learning approach was found helpful to students in remembering the concepts of elements and compounds. Increased student interactions and participation were observed, and the game seemed to be more engaging cognitively and collaboratively. A study by Höft and Bernholt (2019) also confirmed that “school science activities, which provide the potential for cognitively activating learning opportunities, could enhance the relationship between students’ interest and conceptual understanding” (p.1). These findings prove the need to encourage students to play with/manipulate the objects because they will tend to learn more when they get involved cognitively, physically, and socially.

### 4.4 Alkhimia

The “Alkhimia” is a computer-based learning program for the lower secondary school chemistry curriculum. The game was administered by Chee and Tan (2012) to two high-ability classes, of which one class of 40 was assigned to the experimental group while the other class of 38 was assigned to the control group. An attitudinal survey was also administered before and after the Alkhimia intervention. Based on the conceptual understanding of the effective separation task, the study revealed that students who were exposed to the intervention outperformed their counterparts in the control group. The intervention was beneficial as it shifted the traditional classroom culture to a community of inquiry characterized by critical and interrogative thinking.

### 4.5 Board-game

The results showed that the board game effectively reconstructed students’ knowledge, demonstrating that the games can serve as a useful pedagogical tool in higher education (Antunes et al., 2012).

### 4.6 Card game and bioplastic synthesis activity

A series of games and hands-on activities relating to the design, synthesis, and function of materials and polymers were designed by Clapson et al. (2020) as complementary learning resources for use in a second-year materials chemistry course for engineering students. The fact that all materials incorporated into the game activities or hands-on guided experimental activities are household items (Clapson et al., 2020), these games and activities helped students to understand the relationships between chemical structure and observable materials properties. Likewise, some activities leveraged a friendly competitive atmosphere to boost engagement and learning.

### 4.7 Card games and computer games

Rastegarpour and Marashi (2012) found that teacher-made instructional card game and computer games are effective tools for learning chemistry concepts. These games are influential in the learning of abstract concepts. They have the prospective to offer chemistry teachers and educators insight in helping students create intangible associations between different topics and promote meaningful learning of chemistry concepts. The results demonstrated that playing games endorsed active learning, concentration, and utilization of trial and error. The authors believe that a well-developed educational game, in addition to its potential for learning and entertainment, can promote interaction between peers.

### 4.8 Chairs!

This game was developed to teach the ring flip of cyclohexane in high school and college organic chemistry classrooms. According to Winter et al. (2016), the ‘*Chairs!’* game was first tested on 41 high school students in 2014, and later on, tested on 50 college students in 2015. The game was found beneficial in both scenarios as it strengthened students' spatial reasoning and improved their conceptual understanding of conformational isomers.

### 4.9 CheMakers

This game involved 47 first-year students in an organic chemistry course. Implementers of this game (Zhang et al., 2021) found that forty-three (43) of these students gave positive feedback on the possibility of playing the game again. Besides promoting higher-order thinking, creativity, and problem-solving skills, CheMakers improved students' confidence in handling difficult questions. Students’ qualitative feedback also indicated that CheMakers was a suitable teaching tool for enhancing discussions and competition. It was further revealed that CheMakers enabled students to memorize content in “a fun, and stress-free manner.” Finally, the authors recommended that while the game was trialed on undergraduate students, it was adaptable to both the ordinary and advanced level chemistry classrooms.

### 4.10 ChemDraw

This game involved nine students (players) and was hosted online using web-conferencing software and implemented through a “molecule madness” tournament. Fontana (2020) found that ChemDraw strengthened students' organic chemistry skills, positively impacted their wellness, and improved social interactions. Despite having been developed for second-semester university students, authors have proposed that the ChemDraw is helpful at all levels of chemistry education. It has also been recognized that this approach played a significant role in students’ mental health that has been threatened by the Covid-19 related news and social distancing rules.

### 4.11 ChemEscape

ChemEscape is a physical adventure game requiring students (players) to solve a series of riddles and puzzles. Clapson et al. (2020) developed four puzzles (Battle boxes) targeting grades 4 to 12 (5000 participants) and first-year engineering students (800 participants). Participants indicated that ChemEscape was a suitable learning tool. It enhanced their knowledge application to new settings, strengthened teamwork and problem-solving skills, and enabled their visualization and enactment of scientific ideas. This game also appears to promote the aspirations of Eilks et al. (2013), who advocated for “hands-on and minds-on” student-centered activities in chemistry classrooms. According to the authors of the ChemEscape game (Clapson et al., 2019), efforts to associate student learning outcomes of ChemEscape with Bloom's Taxonomy were underway. It was also stated that authors were still exploring incorporating ChemEscape in large classes at tertiary levels of education.

### 4.12 Chemical nomenclature application

A game-based application named Chemical Nomenclature was developed for Android and IOS, a free-of-charge, dynamic, and easy-to-play game that allows students to review chemical nomenclature (Sousa Lima et al., 2019). Student testing revealed that the game design, content, playability, and usefulness were complementary didactic tools to aid in traditional study. Assessment of student knowledge gains was performed. The results revealed that students who used the game as a complementary tool had higher performance in tests than students who studied nomenclature using only conventional learning methods.

### 4.13 Chemory game

Student motivation was found to increase significantly by Chemory game compared to the traditional lecture format. This game also was found to increase self-study time per week among the students. The failure rate in the final examination was also reduced, mainly because of bonus points that students could receive upon successful participation in the game (Daubenfeld and Zenker, 2015).

### 4.14 Element cycles

The game involved 95 s-year high school students. Although the authors (Pippins et al., 2011) did not manage to compare the results of those who participated in the game with those who did not, they concluded that the participants showed a significant improvement in their retention of essential elements from the average pretest score of 40%–50% in the posttest. It was further stated that the game was adaptable to different chemistry content and grade levels. The game was quite enjoyable, fun, and an effective chemistry teaching and learning tool.

### 4.15 Escape Room

Forty (40) students and four high school and university teachers participated in the “Escape Room Game.” Dietrich (2018) indicated that at least 90% of the survey panel said that the game increased students’ motivation and communication and strengthened their teamwork skills. Furthermore, 67% of the panel indicated that the game promoted active learning among students instead of the traditional classroom environment. The game was found beneficial among students in the classroom and teachers and other technical personnel for team-building.

### 4.16 Game-based approach

Unlike other studies in which a single game has been used as a teaching tool, Wilson and Samide (2014) incorporated various games in teaching analytical chemistry and organic chemistry to undergraduate students. They also found that a game-based approach enabled students to develop deeper thinking and interactive skills on top of having fun. Overall, these authors argued that, unlike most previous studies that have used games in isolation to teach specific content, it was better to employ various games to teach one specific unit. Though taxing in terms of preparation and implementation time, these findings prove that incorporating multiple games into a single teaching unit is bound to give a context appropriate for learners with diverse interests and reasoning abilities.

### 4.17 Ion Hunters

This game was piloted with 22 students who had already taken chemistry lessons and the Science Education program at a public university in Turkey. The game was found to be more engaging, enjoyable, and created fun among the students. Yenikalaycl et al. (2019) indicated that ‘Ion Hunters’ effectively improved students' motivation to learn, which eventually led to an enhanced understanding of anions and cations. The authors further indicated that this game could be played at any level of chemistry education where ions are taught.

### 4.18 Misha and Kosha game-based learnings

Partovi and Razavi (2019) revealed that the educational computer game impacted the academic achievement motivation of elementary students; the experimental group had significantly higher scores for academic achievement motivation than the control group. Since the necessity of using computer-based games in elementary school students was realized, the authors recommended finding a more suitable place in the teachers’ daily lesson plan.

### 4.19 Molebots

Molebots is a first-person shooter game focused on chemical nomenclature. It was piloted with students in the first semester of a general chemistry course in the United States (Gupta, 2019). The game was announced in an online course conducted through the Desire to Learn (D2L) learning management system. The survey results showed that students enjoyed using the game and preferred games over other media—specifically, the textbook was the least preferred learning method.

### 4.20 ORG600 (organic reactions game)

Undergraduate students tested and evaluated the game, and their opinions revealed that they liked to use it as a complementary educational tool to aid their studies (da Silva Júnior et al., 2021). The authors concur that the intuitive and interactive features can allow Chemical Engineering and Chemistry students to review the classroom content and improve their exams’ performance. In their next study, authors intend to assess the learning process by comparing performance and academic achievement from classes that do not use the app in their studies.

### 4.21 Pantomime MisCoAct

Since knowing about misconceptions is of great importance for future chemistry teachers, misconceptions activity “MisCoAct” showed potential to be a fruitful way of consolidating and repeating the most frequently occurring misconceptions. According to Belova and Zowada's (2020) research, the competitive aspect, particularly, leads to increased motivation.

### 4.22 Picture Chem

The game was first trialed with 18 teachers who volunteered to participate. After identifying the strengths, weaknesses, opportunities, and possible threats, the game was administered to 20 first-year students (enrolled in a chemistry class/lab). Kavak and Yamak (2016) revealed that teacher participants appreciated the game, indicating that it was quite motivating, fun, and inexpensive despite the game being challenging. The game was also characterized by visual learning opportunities and was suitable for all age groups and gender. This makes it ideal for lower secondary students as well. Some of the weaknesses that were associated with the game include insufficient time and lack of apparatus. Although the game was seen as adaptable to different situations, teacher participants indicated that teachers who do not embrace inquiry-based learning might find it quite odd to incorporate this game in their teaching activities. In the second phase of the Picture Chem game implementation, a paired samples t-test showed a significant improvement in students’ learning and understanding of standard chemistry laboratory apparatus.

### 4.23 Work-integrated learning (WIL)

Ponikwer and Patel (2021) study has focused on developing a game-based WIL activity that can be conducted on-campus and can create and showcase an array of essential skills that can enhance their employability. This activity focused on evaluating and promoting a new chromatographic column using a range of different marketing mediums. This activity was found effective as it provides the ability to students to showcase a wide range of skills that are often difficult to cover in a chemistry degree program, such as nonscientific communication, video making, and marketing skills.

## 5 Study implications

The fact that more studies are implementing games in the classroom in current years may be explained by the implementation of blended learning. Specifically, Covid-19 ordered teachers and students to adapt to ICT-related media. While digital game-based learning may be perceived as challenging for developing countries in sub-Saharan Africa, which is not fully equipped technologically, it suffices to point out that technology in education has come to stay. Online learning has become paramount in times of crisis, such as the one being experienced world-over (i.e., the COVID-19 lockdown). This is evident by the results of a recent study by Wang and Zheng (2020), conducted on Chinese middle school students to compare teaching approaches that were characterized by digital games, non-digital games, and traditional lectures. They found that game-based learning approaches performed significantly better than the conventional teaching approach. While no significant differences in students’ performance in science were detected, the study revealed that students exposed to digital game-based learning approaches exhibited a higher self-efficacy than those exposed to no-digital game-based learning.

These findings are in no way at variance with those of previous studies. For instance, other scholars (Barko and Sadler, 2013; Vogel et al., 2006) have also postulated that immersive learning environments offered by digital games are characterized by several benefits, including interactive learning experiences, extraneous load reduction, and the mental construction of scientific knowledge, among others. Furthermore, digital game-based learning has also been found to enable learners to have fun while learning. As such, we concur with Mukuka et al. (2021b), who recommended a need for the education systems in sub-Saharan Africa to put up infrastructure that supports digital learning to ensure that students continue learning even in times of calamities like the COVID-19 outbreak that may disrupt the face-to-face physical interactions between the teacher and the learners.

Educators use game-based learning in order to stimulate learners' interest. For instance, Baek et al. (2015) denoted that students get motivated while learning chemistry through games. While the study findings and those of previous studies (e.g., Battersby et al., 2020; Franco-Mariscal et al., 2016; Sousa Lima et al., 2019) suggest that game-based learning is bound to improve students' confidence in the subject matter, Zhang et al. (2021) cautioned that there was not enough evidence that the game could improve students' interest in learning chemistry. This demonstrates a need for future users (teachers and researchers) of the game to incorporate some features that will focus on fostering conceptual understanding and increased motivation in chemistry learning as it is also a potential predictor of students’ achievement. The fact that game-based learning was widely found to be used in high schools and colleges than elementary school. Actually, little children like games, but when it comes to playing games while learning, the situation changes. Students in secondary schools and universities are more mature and can be serious in learning through games more than only enjoying like pupils in primary schools. However, this is a motivational outcome that needs more future research outlook from the game designers and capacity or attitude of children.

The scarcity of ICT-related tools may explain the absence of implementing educational games in Africa. Our literature analysis also demonstrates that non-digital games are more physically engaging than digital games. This could be attributed to the fact that some games like Escape Room (Dietrich, 2018) may involve students using different body parts during the activity. This clearly shows that schools with insufficient digital resources can resort to non-digital games that are likely to engage students physically, socially, and intellectually throughout the learning process. Furthermore, studies conducted by Mukuka et al. (2021a) and Ukobizaba et al. (2021) also found that engaging students physically, socially. They intellectually did not only enhance their mathematical reasoning skills but also improved their problem-solving skills. Therefore, this demonstrates a severe need to employ activity-based learning techniques in chemistry classrooms and other STEM subjects such as mathematics, biology, and physics.

## 6 Conclusion

The literature review shows that most games have been tested in high school and university-level chemistry classrooms. The good thing is that most of these games' developers have indicated that these games could be adapted to the lower levels of chemistry education. The extent to which such approaches address the curriculum requirements equally needs to be explored in future studies. It has been noted that teachers rarely involve games in their teaching. This could be attributed to some reasons, including teachers' inadequate orientation on using the game-based learning approach, lack of resources, and inadequate science classrooms and laboratories. Though challenging in terms of preparation/implementation time, game-based learning has been described as an approach that enhances students’ understanding of science subjects. This calls for a serious need for investing in infrastructure that supports the implementation of game-based learning (both digital and non-digital), especially in the less developed world, where such techniques have not been implemented substantially.

## Declarations

### Author contribution statement

All authors listed have significantly contributed to the development and the writing of this article.

### Funding statement

This work was supported by the African Center of Excellence for Innovative Teaching and Learning Mathematics and Science (ACEITLMS). The project number is ACE II (P151847).

### Data availability statement

The data is uploaded on figshare repository and available at https://doi.org/10.6084/m9.figshare.19229217.v1.

### Declaration of interests statement

The authors declare no conflict of interest.

### Additional information

No additional information is available for this paper.
